# Publisher Correction: nAChRs gene expression and neuroinflammation in APPswe/PS1dE9 transgenic mouse

**DOI:** 10.1038/s41598-021-93288-4

**Published:** 2021-07-06

**Authors:** Chiara D’Angelo, Erica Costantini, Nieves Salvador, Michele Marchioni, Marta Di Nicola, Nigel H. Greig, Marcella Reale

**Affiliations:** 1 Department of Medical, Oral and Biotechnological Sciences, University “G. D’Annunzio”, Via dei Vestini 31, 66100 Chieti, Italy; 2 Department of Molecular, Cellular and Developmental Neurobiology, Instituto Cajal-CSIC, Madrid, Spain; 3 Drug Design and Development Section, Translational Gerontology Branch, Intramural Research Program, National Institute on Aging, National Institutes of Health, Baltimore, MD 21224 USA

Correction to: *Scientific Reports* 10.1038/s41598-021-89139-x, published online 06 May 2021

The original version of this Article contained errors in the author list, where the author first and last names were reversed. The correct author names appear below: Chiara D’Angelo, Erica Costantini, Nieves Salvador, Michele Marchioni, Marta Di Nicola, Nigel H. Greig and Marcella Reale. The original Article and accompanying Supplementary Information file have been corrected.

